# Structural and evolutive features of the *Plinia phitrantha* and *P. cauliflora* plastid genomes and evolutionary relationships within tribe Myrteae (Myrtaceae)

**DOI:** 10.1590/1678-4685-GMB-2021-0193

**Published:** 2022-01-31

**Authors:** Lilian de Oliveira Machado, Valdir Marcos Stefenon, Leila do Nascimento Vieira, Rubens Onofre Nodari

**Affiliations:** 1 Universidade Federal de Santa Catarina, Departamento de Fitotecnia, Programa de Pós-graduação em Recursos Genéticos Vegetais, Florianópolis, SC, Brazil.; 2 Universidade Federal do Paraná, Departamento de Botânica, Curitiba, PR, Brazil.

**Keywords:** Jaboticaba, phylogenomic analysis, plastome evolution

## Abstract

*Plinia phitrantha* and *P. cauliflora* are Myrtaceae species with recognized horticultural and pharmacological potential. Nevertheless, studies on molecular genetics and the evolution of these species are absent in the literature. In this study, we report the complete plastid genome sequence of these species and an analysis of structural and evolutive features of the plastid genome within the tribe Myrteae. The two plastid genomes present the conserved quadripartite structure and are similar to already reported plastid genomes of Myrteae species concerning the size, number, and order of the genes. A total of 69-70 SSR loci, 353 single nucleotide polymorphisms, and 574 indels were identified in *P. phitrantha* and *P. caulifora*. Observed evolutive features of the plastid genomes support the development of programs for the conservation and breeding of *Plinia*. The phylogenomic analysis based on the complete plastid genome sequence of 15 Myrteae species presented a robust phylogenetic signal and evolutive traits of the tribe. Ten hotspots of nucleotide diversity were identified, evidence of purifying selection was observed in 27 genes, and relative conservation of the plastid genomes was confirmed for Myrteae. Altogether, the outcomes of the present study provide support for planning conservation, breeding, and biotechnological programs for *Plinia* species.

Several studies have demonstrated that plastid genome sequences are quite useful tools for phylogenetic inferences (de Santana Lopes et al., 2018a; Machado et al., 2017, 2020; Nagel et al., 2020) and investigation of evolutionary events (de Santana Lopes *et al*., 2018b, 2019, Stefenon *et al*. 2019). Plastid genomes of some species of tribe Myrteae (Myrtaceae) have been sequenced (Eguiluz *et al*., 2017a, 2017b; Machado *et al*., 2017, 2020; Rodrigues et al., 2020) and may assist in understanding the evolution and solving taxonomic uncertainties within this tribe. The taxonomy of Myrteae has been considered particularly difficult (Vasconcelos et al., 2017) due to morphological conservatism, relatively homogeneous flowers, and the rarity of single diagnostic characters for individual clades (Lucas et al., 2019).

The scarcity of genetic and genomic resources for species of minor economic importance but with high cultural and ecological value is one of the main struggles towards conserving genetic resources and evolutionary history understanding. This is particularly true for Myrteae, which has a few species with significant genetic and genomic resources available. Aiming at generating novel genomic resources for tribe Myrteae, we sequenced, assembled, and characterized the complete plastid genomes of *Plinia phitrantha* (Kiaersk.) Sobral and *Plinia cauliflora* (Mart.) Kausel, two fruit species with high economical potential. 

Plastids were isolated from young and fresh leaves from single adult trees according to Machado et al. (2017). Plastid-enriched DNA was isolated from the purified plastids using the CTAB protocol. Plastid DNA was sequenced on an Illumina MiSeq Sequencer platform. The paired-end reads were *de novo* assembled using the CLC Genomics Workbench v8.0.1. The plastid genomes annotation was performed using the DOGMA software (Wyman *et al*. 2004) and tRNAscan-SE (Schattner et al., 2005). Inverted Repeat regions (IRs) were identified using REPuter (Kurtz and Schleiermacher 1999). The circular cpDNA maps were built using the OrganellarGenomeDRAW program (Lohse et al., 2013). 

The plastid genomes of *P. phitrantha* and *P. cauliflora* were compared based on structural features, gene content, single nucleotide polymorphisms (SNPs), insertions-deletions (indels), synonymous *(Ks)* and nonsynonymous *(Ka)* substitution rates, and the Ka/Ks ratio.

Boundaries and sizes of the IRa, IRb, SSC, and LSC regions, hotspots of sequence divergence, SSR loci, relative synonymous codon usage (RSCU), potential RNA editing sites, and phylogenomic analysis were investigated for the two newly sequenced and 13 further plastid genome sequences of species from tribe Myrteae (Table S1). The order of the genes of four *Plinia* species and *Rhodomyrtus tomentosa* (Myrtaceae) was compared through the alignment of plastid genomes using the progressive MAUVE strategy (Darling *et al*., 2004). 

The plastid genomes of *P. phitrantha* (GenBank ID KY392759) and *P. cauliflora* (GenBank ID KX527622) presented 158,918 bp and 159,095 bp in length, respectively (Figure 1a; Table 1). The size of the long single copy region (LSC), the short single copy region (SSC), the IRa and IRb regions are presented in Table 1. The total GC content of the genome was 36.96% for *P. phitrantha* and 36.97% for *P. cauliflora*. The regions LSC, SSC, and IR presented 34.8%; 30.8%, and 42.7% of the GC content respectively. These sizes are similar to those already sequenced for other species of tribe Myrteae (Eguiluz et al., 2017a, 2017b; Machado et al., 2017, 2020; Rodrigues et al., 2020; Table S1). A total of 112 different genes were identified: 78 protein-coding, 30 tRNA genes, and four rRNA genes (Table 1). 

**Figure 1 -:**
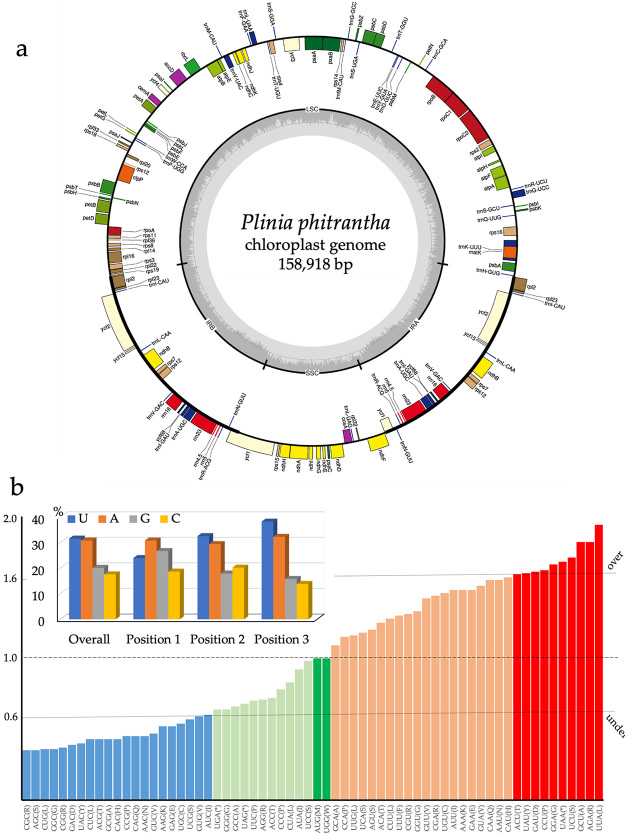
(a) Gene map of *Plinia phitrantha* plastid genome. Genes drawn inside the circle are transcribed in the clockwise direction, and genes drawn outside are transcribed in the counterclockwise direction. Different functional groups of genes are color-coded. The darker gray in the inner circle corresponds to the GC content, while the lighter gray corresponds to the AT content. LSC: large single copy, SSC: small single copy, IRa, and IRb: inverted repeat A and B, respectively. The gene map of *Plinia cauliflora* plastid genome is physically identical to *P. phitrantha*. (b) Relative synonymous codon usage (RSCU) analysis for *P. phitrantha* and *P. cauliflora.* The output of the analysis was equivalent for both species. The upper dotted line (over) corresponds to RSCU = 1.6 (over-representation limit), while the lower dotted line (under) corresponds to RSCU = 0.6 (under-representation limit). RSCU = 1.0 (dashed line) means the absence of bias in codon usage. The inset represents the frequency of each base overall and in each specific codon position.

**Table 1 -:** Summary of the characteristics of the plastid genomes newly sequenced in this study.

	*Plinia phitrantha*	*Plinia cauliflora*
GenBank ID	KY392759	NC039395
Total number of mapped reads	20,991,775	89,204,034
Average reads length (bp)	280.91	198.48
Mapped reads aligned (bp)	258.87	217.55
Deep genome coverage^$^	130´	450´
cpDNA genome size (bp)	158,918	159,095
LSC size in bp	88,204	88,162
SSC size in bp	18,462	18,615
IR size in bp	26,126	26,159
PCG genes	78	78
tRNA genes	30	30
rRNA genes	4	4
Genes duplicated by IR	20	20
Genes with introns	18	18
Overall GC content (%)	36.96	36.97
GC content in the LSC (%)	34.8	34.8
GC content in the SSC (%)	30.8	30.8
GC content in the IR (%)	42.7	42.7

cp: chloroplast; PCG: protein-coding gene; ^$^nº of sequenced bases/estimated plastid genome size

The RSCU analysis revealed the same pattern for *P. phitrantha* and *P. cauliflora* (Figure 1b). A high proportion of synonymous codons presenting the nucleotides A (30.6%) or U (31.7%) in the third position was observed in both species. The shared pattern of codon usage bias suggests the same evolutionary path for these species and the likely absence of barriers for interspecies hybridization. Thirty codons presented RSCU values higher than 1.0, meaning they are being used more often than expected, while 32 codons are being used less frequently than expected (RSCU < 1.0). Following Barbhuiya *et al*. (2020), a total of 21 codons are under-represented (RSCU < 0.60), and 10 codons are over-represented (RSCU > 1.6). 

A total of 353 single nucleotide polymorphisms (SNPs) and 574 indels ranging from one to 17 bases between *P. phitrantha* and *P. caulifora* were identified. The SNPs were located within 33 genes, seven introns, and 55 intergenic regions (Figure 2). 

**Figure 2 -:**
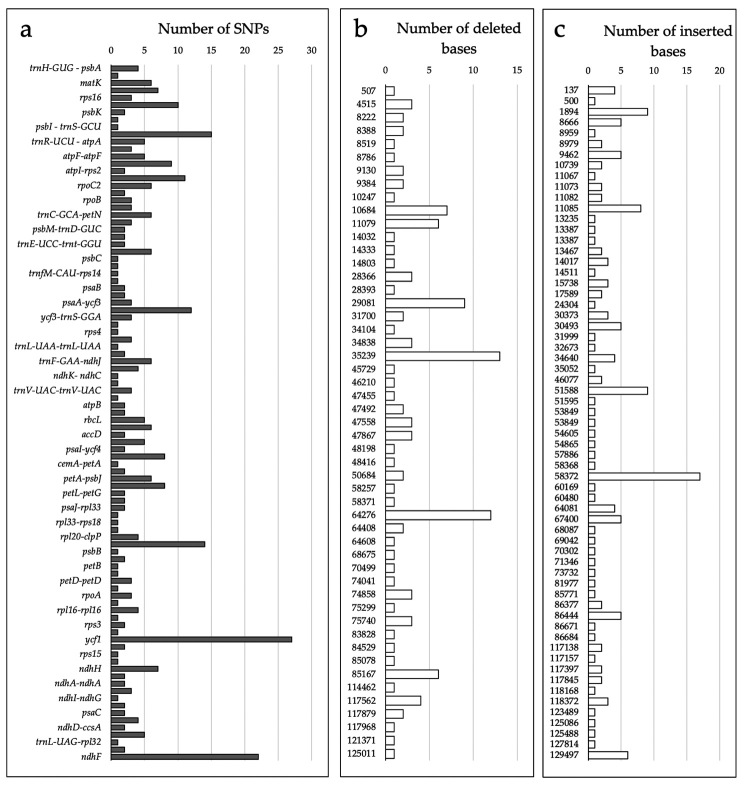
Distribution of SNPs and indels prospected in the comparison between the plastid genomes of *P. phitrantha* and *P. cauliflora*. **(a)** Number of SNPs in different regions of the plastid genomes. **(b)** Number of deleted bases in the plastid genome of *P. phitrantha* in comparison to *P. cauliflora*. **(c)** Number of inserted bases in the plastid genome of *P. phitrantha* in comparison to *P. cauliflora*. The numbers in the y-axis of **b** and **c** correspond to the genomic position, starting from the IRa region in an anti-clock direction.

The highest nonsynonymous rate was observed in the *petB* gene (Ka = 0.7376), while the *rbcL* gene had the highest synonymous rate (Ks = 0.8675). No changes were observed in the synonymous or nonsynonymous rates in 44 genes*.* Evidence of purifying selection (Ka/Ks ratio < 1.0) was observed in three genes associate with the small subunit of the ribosome (*rps3, rps4,* and *rps12*), two with the large subunit of the ribosome (*rpl23* and *rpl32*), two with the RNA polymerase subunits (*rpoA* and *rpoC1*), three with the ATP synthase gene (*atpA, atpB,* and *atpE*), five with the NADH dehydrogenase (*ndhA, ndhD, ndhF, ndhH,* and *ndhK*), two with the cytochrome b/f complex (*petA* and *petB*), two with the photosystem I (*psaA* and *psaB*), four with the photosystem II (*psbA, psbB, psbC,* and *psbK*), two with unknown functions (*ycf1* and *ycf2*), in the cytochrome c biogenesis (*ccsA*), in the acetyl-CoA carboxylase (*accD*), and the maturase (*matK*) genes. No evidence of positive selection (Ka/Ks ratio > 1.0) was observed. 

Using the plastid genome of *Arabidopsis thaliana* as a reference, a total of 459 RNA editing sites were predicted for *P. phitrantha* and 458 for *P. cauliflora*. No silent putative editing site was predicted. Most of the editing sites (ca. 98.3%) were shared between species. The correspondence of these sites in *P. phitrantha* and *P. cauliflora* suggests a lack of incompatibility for interspecific hybridization since RNA editing affects plastid gene expression and, therefore, could be involved in nuclear-cytoplasmic incompatibility in interspecific hybrids (Pacheco *et al*., 2020).

Concerning the SSR loci identified in the plastid genomes, the A/T motif was the most frequent. The monomer motif C/G was found only in *E. selloi* and *P. dioica*, TAT/ATA only in *C. xanthocarpa*, ACT/AGT only in *R. tomentosa*, and AAT/ATT in *E. selloi*, *P. clatteyanum*, and *P. guajava*. The outgroup species (*Corymbia eximia, Allosyncarpia ternata*, and *Lagerstroemia fauriei)* presented a similar pattern of motifs occurrence (Figure S1). 

The phylogenomic analysis (Figure 3) based on the complete plastid sequences using *Corymbia eximia* (Eucalypteae, Myrtaceae; NC022409)*, Allosyncarpia ternata* (Eucalypteae, Myrtaceae; NC02243), and *Lagerstroemia fauriei* (Myrtales, Lythraceae; NC029808) as outgroups returned an overall topology congruent to that based on nuclear and plastid genes. The *Plinia* group (BP = 100%) is a sister of the group formed by all other Myrteae South American species. *Eugenia* and *Psidium* also formed monophyletic groups (BP = 100%). The *Eugenia* group is related to *Myrcianthes pungens*, while *Acca sellowiana* is a sister of *C. xanthocarpa* (BP = 99%). The Australasian Myrteae *R. tomentosa* is basal to the South American species. This result supports previously published phylogenetic studies of tribe Myrteae based on nuclear and plastid DNA sequences (Vasconcelos et al., 2017). 

**Figure 3 -:**
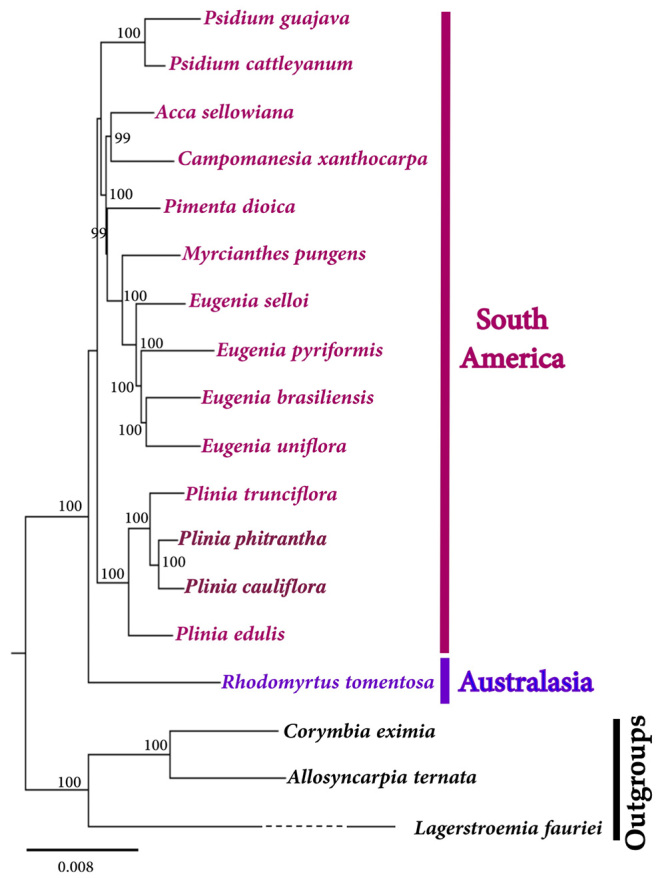
Maximum likelihood phylogenomic relationship among species of tribe Myrteae based on whole plastid genome sequences. *C. eximia, A. ternata,* and *L. fauriei* were used as outgroups. Numbers at the nodes are bootstrap support after 1000 permutations. The branch length of *L. fauriei* was shortened in the figure (dashed line).

In all species, the IRb/LSC border is located between genes *rpl2* and *rps19*, while the *ycf1* gene highlights the IRa/SSC junction, crossing across both genomic regions (Figure S2). The analysis of local collinear blocks (LCBs) architecture within the plastid genomes of *Rhodomyrtus tomentosa* (the earliest divergent species within Myrtaeae) and the four *Plinia* species revealed two main LCBs with four short regions with diverse distribution or occurrence among these plastomes (Figure S2).

The sliding window analysis generated a consensus of 169,244 bp and revealed 10 hotspots of nucleotide divergence with π > 0.040, a value five-fold higher than the overall nucleotide diversity (π = 0.00725) estimated for the whole plastid genome. Six of these hotspots are in intergenic spacer regions (IGS), two are in intronic regions, one corresponds to a gene (*ndh*K), and one includes part of an intron and part of the second exon of the *clp*P gene (Figure S3). Considering the puzzling taxonomic classification of *Plinia* species based on morphological traits (dos Santos *et al*. 2021), these polymorphic regions, the SNPs, and the indels identified between *P. phitrantha* and *P. caulifora* are promising sources of taxonomic markers for *Plinia* species.

Even an inversion observed in the SSC region of the plastid genomes of *P. phitrantha, P. edulis,* and *E. pyriformis* is not an irregular event (Walker et al., 2015). Chloroplast DNA within individual plants can exhibit a form of heteroplasmy in which the plastome exists in two equimolar states (inversion isomers) that differ in the relative orientation of the SSC region (Palmer, 1983; Walker *et al*., 2015). Finally, the conservation of the plastid genomes is corroborated by the analysis of LCBs. Only four small regions differ among *Plinia* species and *R. tomentosa*, whereas the main CLBs are conserved through the five plastid genomes (Figure S4).

Patterns of structural conservation and synteny among six plastid genomes of Myrteae species were also reported by Rodrigues et al. (2020). Here we show that such preserved patterns were shared with *R. tomentosa*, an earlier divergent species within Myrtaceae (Vasconcelos et al., 2017). 

## Supplementary material

The following online material is available for this article:

Table S1 **-**
Comparative summary of some features of the Myrtaceae plastid genomes included in this study.

Figure S1 - (a) Distribution of the SSR loci within the plastid genome of *P. phitrantha* and *P. cauliflora*.

Figure S2 - Boundaries at the junctions of the LSC, IRb, IRa, and SSC regions of the plastid genomes of South American Myrteae species.

Figure S3 - Sliding window analysis of the plastid genomes of 15 Myrteae species.

Figure S4 - Multiple plastid genome alignment with four *Plinia* species and *R. tomentosa*.
