# Supplementary material for: Structural and evolutive features of the Plinia phitrantha and P. cauliflora plastid genomes and evolutionary relationships within tribe Myrteae (Myrtaceae)
Source: Genet Mol Biol. 2022 Jan 31;45(1):e20210193. doi: 10.1590/1678-4685-GMB-2021-0193 (PMC8805445; doi:10.1590/1678-4685-GMB-2021-0193)
Supplement: Table S1 - [file 1415-4757-GMB-45-1-e20210193-s1.pdf]

Genetics and Molecular Biology
https://doi.org/ 10.1590/1678-4685-GMB-2021-0193

Suplementary material to "Structural and evolutive features of the *Plinia phitrantha* and *P. cauliflora* plastid genomes and evolutionary relationships within tribe Myrteae (Myrtaceae)

Table S1. Comparative summary of some features of the Myrtaeae plastid genomes included in this study.

	Size	PCG	tRNA	rRNA	%GC overall	Pseudogenes	Reference
P. phitrantha	158,918	78	30	4	36.96	2	This study
P. cauliflora	159,095	78	30	4	36.97	2	This study
P. edulis	159,631	78	30	4	36.93	1	Rodrigues et al. 2020
P. trunciflora	159,512	78	30	4	37.00	3	Eguiluz et al. 2017
E. uniflora	158,445	78	30	4	37.00	3	Eguiluz et al. 2017
E. brasiliensis	158,251	78	30	4	36.95	1	Rodrigues et al. 2020
E. pyriformis	158,569	78	30	4	37.01	1	Rodrigues et al. 2020
E. selloi	157,683	78	30	4	37.04	1	Rodrigues et al. 2020
M. pungens	159,239	78	30	4	36.94	1	Rodrigues et al. 2020
P. dioica	158,984	78	30	4	--	--	NCBI GenBank
C. xanthocarpa	158,131	77	30	4	36.98	4	Machado et al. 2020
A. sellowiana	159,370	78	30	4	37.00	3	Machado et al. 2017
P. guajava	158,841	78	30	4	37.00	1	Jo et al. 2016
P. cattleyanum	159,088	78	30	4	37.05	1	Rodrigues et al. 2020
R. tomentosa	156,129	84	37	4	37.10	3	Huang et al. 2019

GenBank IDs: *Acca sellowiana* (KX289887), *Campomanesia xanthocarpa* (KY392760), *Eugenia uniflora* (KR867678), *Plinia trunciflora* (KU318111), *Pimenta dioica* (KY085891.1), *Psidium guajava* (KX364403), *Rhodomyrtus tomentosa* (MK044696.1), *Eugenia brasiliensis* (MN095407), *Eugenia selloi* (MN095411), *Eugenia pyriformis* (MN095410), *Myrcianthes pungens* (MN095409), *Plinia edulis* (MN095413), *Psidium cattleyanum* (MN095408), *Rhodomyrtus tomentosa* (MK044696.1).
