# Supplementary material for: Structural and evolutive features of the Plinia phitrantha and P. cauliflora plastid genomes and evolutionary relationships within tribe Myrteae (Myrtaceae)
Source: Genet Mol Biol. 2022 Jan 31;45(1):e20210193. doi: 10.1590/1678-4685-GMB-2021-0193 (PMC8805445; doi:10.1590/1678-4685-GMB-2021-0193)
Supplement: Figure S2 - [file 1415-4757-GMB-45-1-e20210193-s3.pdf]

Genetics and Molecular Biology
https://doi.org/ 10.1590/1678-4685-GMB-2021-0193

Suplementary material to "Structural and evolutive features of the *Plinia phitrantha* and *P. cauliflora* plastid genomes and evolutionary relationships within tribe Myrteae (Myrtaceae)

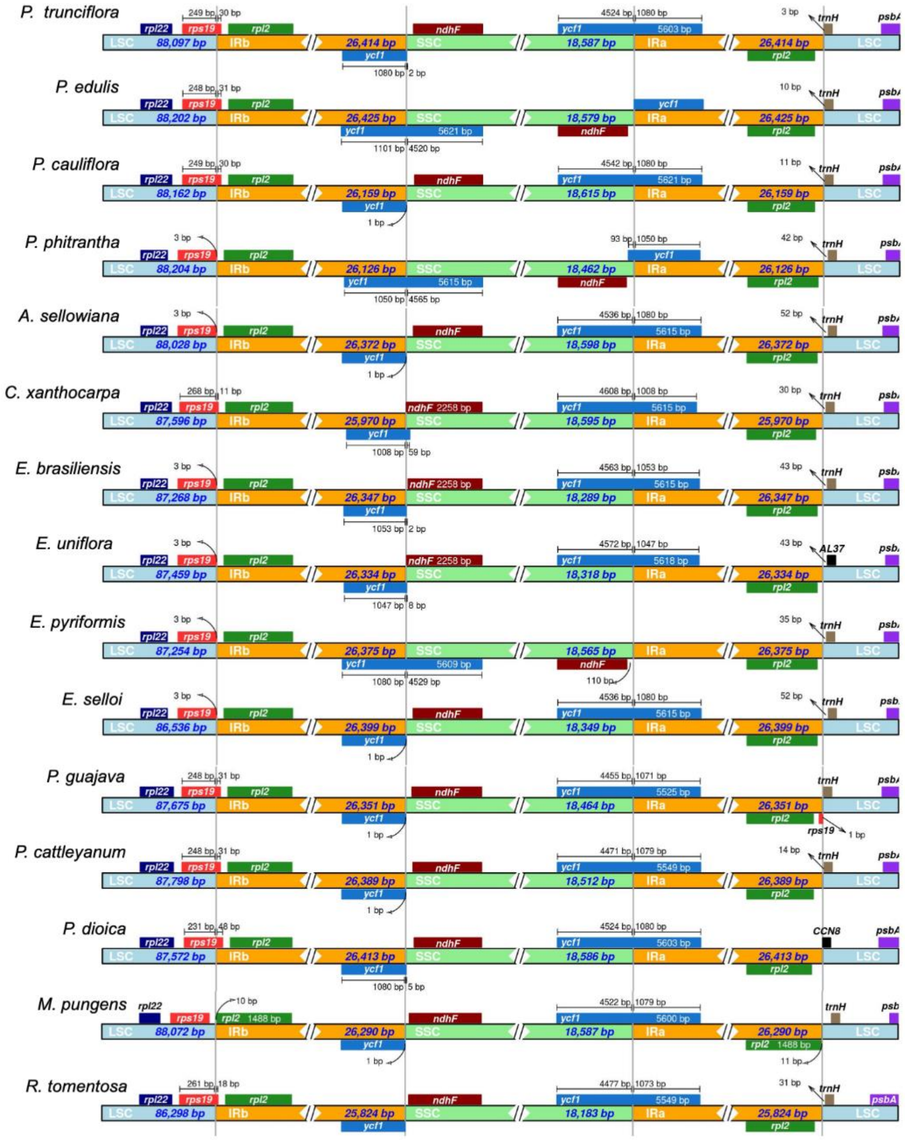

Figure S2 - Boundaries at the junctions of the LSC, IRb, IRa, and SSC regions of the plastid genomes of South American Myrteae species.
