# Supplementary material for: Structural and evolutive features of the Plinia phitrantha and P. cauliflora plastid genomes and evolutionary relationships within tribe Myrteae (Myrtaceae)
Source: Genet Mol Biol. 2022 Jan 31;45(1):e20210193. doi: 10.1590/1678-4685-GMB-2021-0193 (PMC8805445; doi:10.1590/1678-4685-GMB-2021-0193)
Supplement: Figure S3 - [file 1415-4757-GMB-45-1-e20210193-s4.pdf]

Genetics and Molecular Biology
https://doi.org/ 10.1590/1678-4685-GMB-2021-0193

Suplementary material to "Structural and evolutive features of the *Plinia phitrantha* and *P. cauliflora* plastid genomes and evolutionary relationships within tribe Myrteae (Myrtaceae)

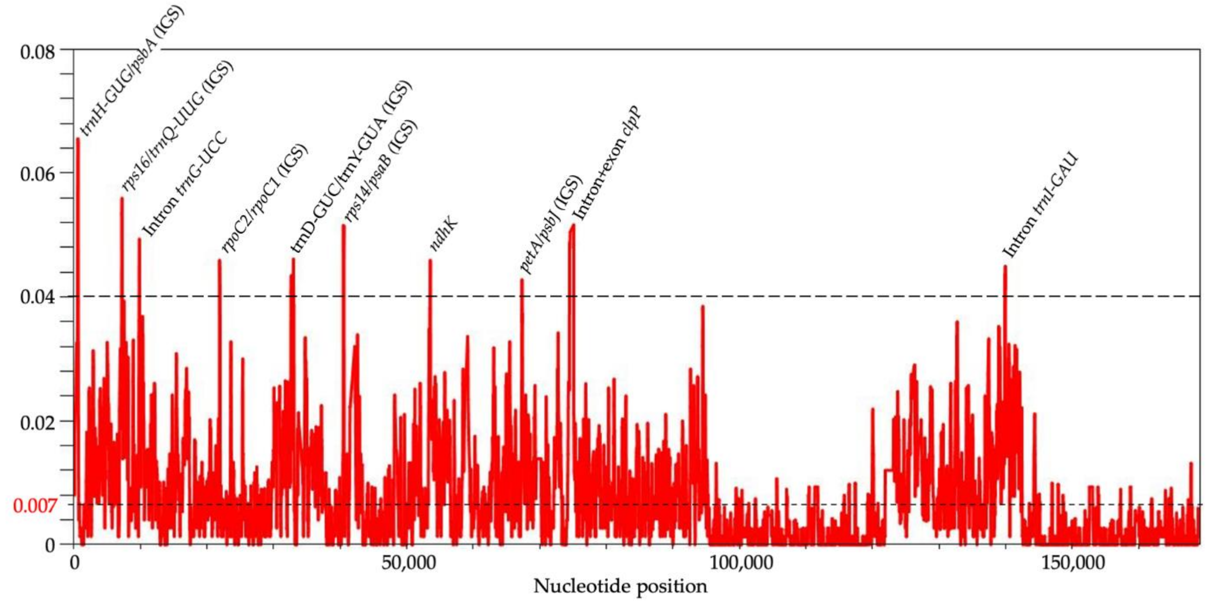

Figure S3 - Sliding window analysis of the plastid genomes of 15 Myrteae species. The dashed line at π = 0.007 corresponds to the estimated overall nucleotide diversity and the dashed line at π = 0.04 is the threshold chosen for the analysis (five-fold higher than the overall nucleotide diversity).
